# Oral *Escherichia coli* expressing IL-35 meliorates experimental colitis in mice

**DOI:** 10.1186/s12967-018-1441-7

**Published:** 2018-03-20

**Authors:** Baoren Zhang, Yi Liu, Xu Lan, Xiaoxi Xu, Xiaoning Zhang, Xiang Li, Yiming Zhao, Guang Li, Caigan Du, Shanzheng Lu, Hao Wang

**Affiliations:** 1 Department of General Surgery, Tianjin Medical University General Hospital, 154 Anshan Road, Heping District, Tianjin, 300052 China; 2 Tianjin General Surgery Institute, Tianjin, China; 3 Department of Genetics, College of Basic Medical Sciences, Tianjin Medical University, Tianjin, China; 4 Department of Endocrinology, Tianjin Medical University General Hospital, Tianjin, China; 5 Department of Urologic Sciences, The University of British Columbia, Vancouver, BC Canada; 6 Immunity and Infection Research Centre, Vancouver Coastal Health Research Institute, Vancouver, BC Canada; 7 Department of Anorectal Surgery, People’s Hospital of Hunan Province, First Affiliated Hospital of Hunan Normal University, Changsha, Hunan China

**Keywords:** Colitis, IL-35, Mice, *Escherichia coli*, Anti-inflammatory

## Abstract

**Background:**

Ulcerative colitis (UC) is a type of inflammatory bowel disease (IBD) characterized by chronic inflammation of colon. It is commonly believed that the imbalance of immune system and overwhelming production of cytokines are involved in the pathogenesis of UC. Recent studies demonstrated that interleukin-35 (IL-35), a key player in the regulation of inflammation, has been identified as potential therapeutic target to treat UC. However, conventional intravenous administration is costly and inconvenient. The present study was designed to establish a novel IL-35 delivery system and investigate its therapeutic effects on dextran sulfate sodium (DSS)-induced experimental colitis in mice for the first time.

**Methods:**

An engineered *Escherichia coli* (*E. coli/IL*-*35*) expressing IL-35 was constructed. Adult male BALB/c mice randomly got the oral administration of *E. coli/IL*-*35*, empty plasmid-transformed *E. coli* (*E. coli0*) or PBS for treatment following ingestion of 3% DSS solution for 5 days. Normal mice were used as control group. Colonic and splenic tissues were collected on day 10 post-DSS-induction. Clinical signs, disease activity index (DAI), pathological and immunohistological changes, cytokine profiles and cell populations were evaluated.

**Results:**

Intragastric administration of *E. coli/IL*-*35* effectively protected the colitis mice from DSS assimilation including weight loss and colon shortening. Pathological analysis showed significantly lower DAI score and much less intra-colon infiltration of neutrophils and CD3^+^ cells in the IL-35 treated group. Moreover, *E. coli/IL*-*35*-treated mice demonstrated much less CD4^+^ IL-17A^+^ Th17 cells and a higher level of CD4^+^CD25^+^Foxp3^+^ Tregs in spleen and mesenteric lymph nodes, as well as increased colon and serum level of IL-10 and IL-35 and decreased levels of IL-6.

**Conclusions:**

Our study showed that *E. coli/IL*-*35* as a novel oral IL-35 delivery system alleviated inflammatory damage of colonic tissue in the colitic mice. Genetic therapeutic strategies using engineered *E. coli* encoding immunoregulatory cytokines may provide a potential approach for the treatment of IBD.

## Background

Ulcerative colitis (UC) is a subtype of inflammatory bowel disease (IBD), characterized by chronic inflammation of the bowel with periods of exacerbation and remission of abdominal pain, diarrhea, purulent stools and relapses [1]. UC is a public problem among individuals of European descent living in wealthy Western countries, while, recent population-based and referral center cohorts have shown a rising incidence and prevalence in Asia [2, 3]. UC featured by ulceration and inflammation in the mucosa and submucosa of the large intestine is considered to be related with overly aggressive T cell-mediated immune responses to intestinal microbiota components in genetically susceptible hosts, with disease initiated and reactivated by environmental triggers [4, 5]. Currently, the pharmaceutical treatments for UC include glucocorticosteroids, immunosuppressive agents and emerging biologic therapy (anti-TNF-α monoclonal antibody). However, there remains a significant cohort of patients with refractory or relapsing disease those who cannot tolerate currently available medical therapy. Also, existing therapeutic options carry an increased risk of infective and malignant diseases [6].

Cytokines, soluble mediators of the inflammatory response secreted by a myriad of cells, take part in the immune reaction. Interleukins are key constituents of the cytokine profile found in the intestinal mucosa of IBD patients and thus have been identified as potential future therapeutic targets [7]. IL-35, composed by heterodimerization of 2 subunits Epstein–Barr virus-induced gene 3 (EBI3) and the IL-12 p35 subunit (IL-12A), has immunosuppressive effects mediated through regulatory T (Tregs) and B cells [8]. Recent studies also demonstrated the anti-inflammatory properties of IL-35 in facilitating the suppressive Treg cells [8, 9], and restricting inflammatory Th17 cells [10–12], making it a promising candidate for treating UC. However, systemic administration of such molecules has several drawbacks including short bioactivity, systemic toxicity, and high cost. Thus, searching for a more effective and economic therapeutic strategy is warranted.

Treatment through regulating intestinal flora attracted much attention in recent years. Traditional probiotics showed limited effectiveness in the treatment of UC, whereas *E. coli* Nissle maintains remission and possibly reduce active inflammation [13]. As the proximal colon exposes to luminal bacteria, colonic mucosa delivery of IL-35 was achieved by oral administration of an invasive and non-pathogenic *E. coli*. In the current study, we constructed a vector of pET-28a(+)-IL35 and used it to design an recombinant *E. coli* (*E. coli/IL*-*35*), aimed to provide therapeutic levels of IL-35 directly in the colon.

## Methods

### Construction of recombinant IL-35 expressing *E. coli/IL*-*35*

pET-28a(+) vector was obtained from Cwbiotech, China. The sequence of IL-35 was a generous gift from Prof. Jiyu Ju (Weifang Medical University, Shandong). The IL-35 segment was gained using *Eco*I/*Xho*I double digestion and subcloned into pET-28a(+) vector linearized to construct pET-28a(+)-IL35 recombinant plasmid, which was transformed into *E. coli* strain BL21(DE3) to get *E. coli/IL*-*35*. The same strain carrying empty-load vector (pET-28a(+)) was used as *E. coli/0*. The expression of the exogenous protein was induced by the addition of 1 mM Isopropyl-β-d-thiogalactopyranoside (IPTG) for 4 h at 37 °C post cultured overnight in LB-medium. Then cells were harvested by centrifugation at 7000×*g* for 10 min at 4 °C, and the obtained pellets were assessed by sodium dodecyl sulfate–polyacrylamide gel electrophoresis (SDS-PAGE) and Western blotting to confirm the expression of IL-35 by anti-IL-12A (Abcam, China).

### Animals

The entire experiment was approved by the Animal Care and Use Committee of Tianjin Medical University (China) according to the Chinese Council on Animal Care guidelines. The male SPF BALB/c mice (Aoyide Co., Tianjin, China) aged 6–8 weeks and weighing 18 ± 2 g. They were housed in comfortable cages at Animal Care Facility of Tianjin General Surgery Institute and allowed to acclimatize to standard lighting, and temperature conditions with food and water freely available before the experiment were performed.

### Experimental groups

The mice were randomly divided into four groups (n = 10 mice per group) as follows: normal group, DSS group, DSS + *E. coli0* group, DSS + *E. coli/IL*-*35* group. DSS-induced colitis model was established in mice according to Kihara et al. [14]. Colitis was induced in 30 mice by free access to 3% DSS solution for 5 days followed by additional 5 days with drinking water without DSS. Mice in DSS group were given 0.2 mL PBS once a day through an oral-gastric tube during the last 5 days. In the DSS + *E. coli/IL*-*35* group, mice were treated with 0.2 mL of PBS suspensions (1 × 10^10^ CFU/mouse/day) of *E. coli/IL*-*35* induced with 1 mM IPTG for 4 h, while those were given the same amount of PBS containing *E. coli0* as control in DSS + *E. coli0* group. All mice were sacrificed by cervical dislocation and examined as described below after fast for 6 h.

### Assessment of inflammation severity

The degree of inflammation in mice was comprehensively assessed by daily disease activity index (DAI) and length of the colon. Briefly, DAI was adopted based on the scoring system of Murthy et al. [15], which represents the sum of scores for weight loss, stool consistency and rectal bleeding divided by three. The colon (from the ileocecal junction to the anus) was collected and the length of it was measured. Then the colon was cutted longitudinally and washed by cold saline, and the colonic contents were removed.

### Histological evaluation

Colon samples were cleaned with saline, fixed in 10% neutral buffered formalin, embedded in paraffin and cut into 5 μm sections. Specimens were dewaxed, hydrated and stained with standard hematoxylin and eosin (H&E) to examine pathological changes in a blinded fashion. The colitis score was used to determine the extent of the inflammation based on a previously published grading system [16], which according to the criteria (a) inflammation severity: 0 (none), 1 (slight), 2 (moderate), 3 (severe); (b) depth of injury: 0 (none), 1 (mucosal), 2 (mucosal and submucosal), 3 (transmural); (c) cryp damage: 0 (none), 1 (basal 1/3 damage), 2 (basal 2/3 damage), 3 (crypt lost, only surface epithelium intact), 4 (entire crypt and epithelium lost); (d) percent involvement: 1 (1–25%), 2 (26–50%), 3 (51–75%), 4 (76–100%). All evaluations were performed by observers unaware of the treatment groups.

### Immunohistochemistry

To quantitate the inflammatory cell infiltration, sections were stained with specific antibodies (Abcam, China). Immunohistochemical staining was performed by using anti-Ly6G and CD3 antibodies to detect intracolonic cellular infiltration of neutrophils and CD3^+^ T cells. Endogenous peroxides of colon specimens were blocked with 3% H_2_O_2_ followed by deparaffination and rehydration, and antigen retrieval was processed by heating in the microwave. The primary antibody was at a dilution of 1:100. Sections only incubated with secondary antibodies were used as negative control. After enclosed by 5% bovine serum albumin (BSA), the specimens were stained according to the instructions of Strept Avidin–Biotin Complex (SABC) kit. Stained sections were photographed using an Olympus inverted microscope (Olympus Imaging America, Center Valley, PA).

### Fluorescence-activated cell sorting (FACS) analysis

FACS analysis was used, as previously described [17], to determine the subpopulation of CD4^+^CD25^+^Foxp3^+^Tregs and CD4^+^IL-17A^+^ Th17 in spleen and mesenteric lymph nodes (MLN). Briefly, splenic and MLN single-cell suspensions were prepared with the final concentration of 1 × 10^7^/mL before immunofluorescent staining. After the lymphocytes were gated from the whole population, the CD4^+^ cells were gated by anti-mouse CD4 antibody for both Tregs and Th17 cells. Then, for the Tregs, the population of both positive staining of anti-mouse CD25 and intracellular Foxp3 antibodies in CD4^+^ cells were calculated. And for the Th17 cells, the population of intracellular IL-17A^+^ cells stained with anti-mouse IL-17A antibody were calculated. In addition, the isotype controls of both Tregs and Th17 cells had been done at the same time. All fluorescent-labeled antibodies were purchased from either eBioscience (eBioscience, San Diego, CA) or BioLegend (BioLegend, San Diego, CA).

### Real-time PCR analysis of inflammation-related genes of colon

The transcriptional gene levels of IL-6, IL-10, IL-35 and IL-12 in colon tissues were determined by quantitative real-time PCR instrument. Total RNA was extracted from colon tissue using TRIzol reagent, and two μg RNA was reverse transcribed into cDNA with the QuantiTect Reverse Transcription Kit (Qiagen) using random hexamers. Quantitative real-time PCR was conducted using the TaqMan gene expression assay with a LightCycler 1.5. GAPDH served as an internal control. The sequences of primers used for analysis were designed as follows: GADPH, sense 5′-AGGTCGGTGTGAACGGATTTG-3′, antisense 5′-TGTAGACCATGTAGTTGAGGTCA-3′; IL-12a, sense 5′-GACCTGGACCCTGAGATTGTGAA-3′, antisense 5′-GGTCCCTGTGCAGCACGTTA-3′; IL-10, sense 5′-AGAAGCATGGCCCAGAAATCA-3′, antisense 5′-GGCCTTGTAGACACCTTGGT -3′; IL-6, sense 5′-CCACTTCACAAGTCGGAGGCTTA-3′, antisense 5′-GCAAGTGCATCATCGTTGTTCATAC-3′; EBI3, 5′-GTTCTCCACGGTGCCCTAC-3′, antisense 5′-CGGCTTGATGATTCGCTC-3′; IL-12p40, sense 5′-CCTGTGACACGCCTGAAGAAGATG-3′, antisense 5′-CTTGTGGAGCAGCAGATGTGAGTG-3′.

### Enzyme-linked immunosorbent assay (ELISA)

Serum was prepared and subjected to ELISA to determine levels of IL-10, IL-35 and IL-6 using ELISA kits (Biolgend, http://www.biolegend.com/) according to the protocol provided by the manufacturer. ELISA was performed in triplicate for each sample.

### Statistical analysis

SPSS 17.0 (SPSS Inc., Chicago, USA) was used for the statistical analysis. The enumeration data were performed as mean ± standard deviation (SD). One-way ANOVA was used for multiple-group comparisons and with proper post hoc analysis. A significant difference was defined as p < 0.05.

## Results

### Construction of pET28a(+)-IL-35 and detection of IL-35 expression in *E. coli/IL*-*35* in vitro and vivo

**Fig. 1:**
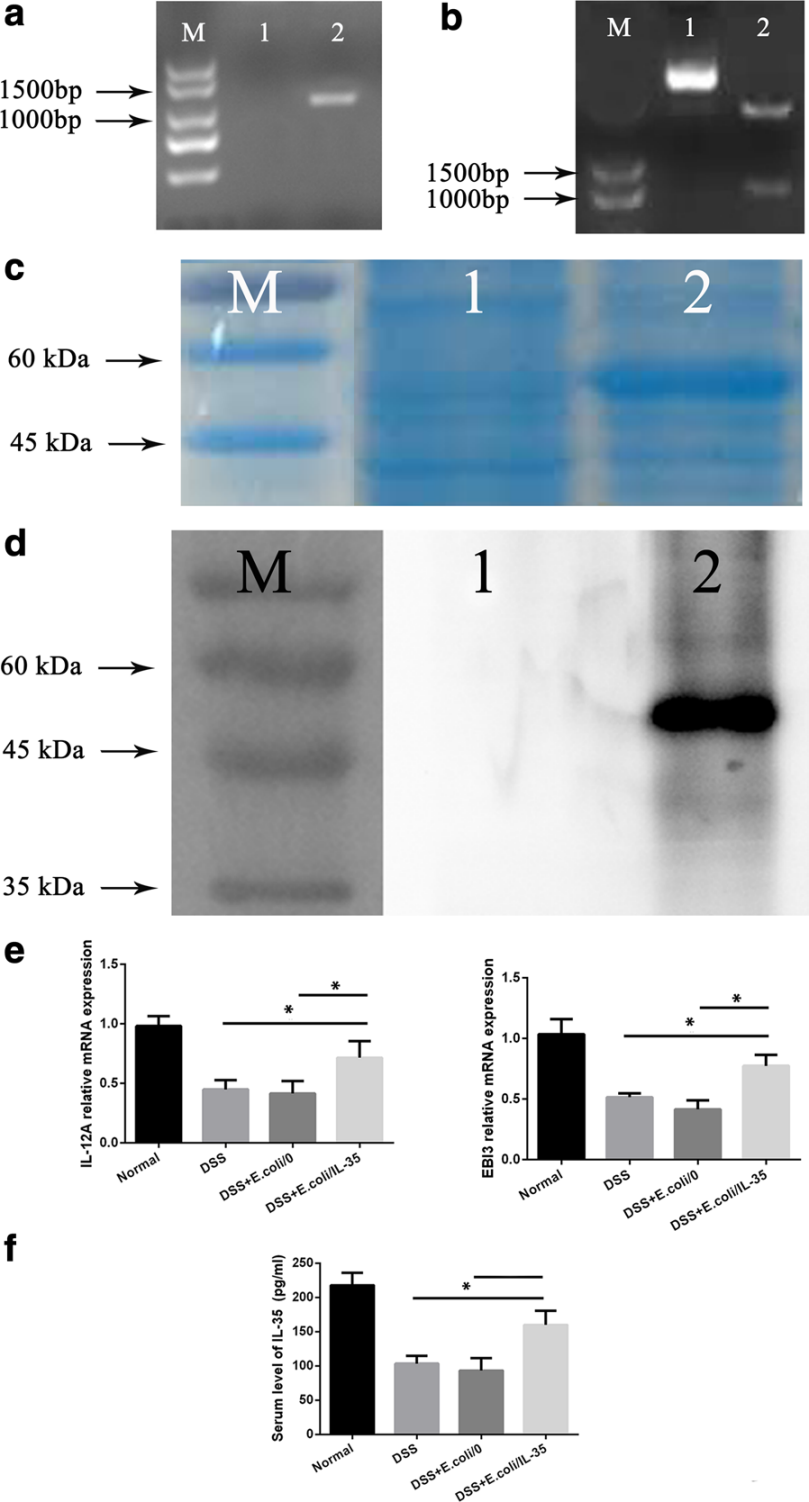
Identification of IL-35 Expression in *E. coli/IL*-*35* in vitro and vivo. **a** Single-colony PCR analysis of recombinant *E. coli/IL*-*35*. Lane M DNA marker DL2000; lane 1 negative control; lanes 2 singlecolony PCR products of recombinant pET28a(+)-IL-35. **b** Double-enzyme digestion of recombinant plasmid. Lane M DNA marker DL2000; lane 1 non-enzyme digestion of recombinant plasmid; lane 2 double-enzyme digestion products of recombinant plasmid. **c** Tricine-SDS-PAGE of the protein expressions in *E. coli/IL*-*35* In lane 1, the strains are transformations of pET28a(+)/IL-35; lane 2 is the empty vector strain. The arrow indicates the dark bands corresponding to the expressed IL-35 protein on Tricine-SDS-PAGE. **d** Western blot analysis of the cell lysate proteins from the lL-35 producer strain *E. coli/IL*-*35* (lane 1) and empty vector strain *E. coli/0* (lane 2). *E. coli/IL*-*35* increased IL-12a and EBI3 mRNA expression in colon tissues (**e**) and the serum concentration of IL-35 (**f**) in DSS-induced colitis mice, n = 10 mice per group. Compared with the DSS group, *p < 0.05; compared with the DSS + *E. coli/0* group, *p < 0.05

The pET28a(+)-IL-35 recombinant plasmid were identified by PCR, EcoR I/XhoI double enzyme digestion and nucleotide sequencing (Fig. 1a, b). The IL-35 expression was analyzed using SDS-PAGE after IPTG treatment. As shown in Fig. 1c, a prominent band of about 56 kDa in the bacteria was transformed with pET-28a(+)-IL35. This band matched the theoretical molecular weight of IL-35 and it was not present in the *E. coli/0*. Consistently, western blot exhibited a strong protein band of 56 kDa in the cell lysate of *E. coli/IL*-*35* instead of *E. coli/0* post induction in Fig. 1d, indicating the successful and stable expression of IL-35. As shown in Fig. 1e, f, after administration of bacterial, the mRNA levels of IL-12A, EBI3 and the serum concentration of IL-35 were significantly increased in the DSS + *E. coli/IL*-*35* group compared to that of in the DSS and DSS + *E. coli/0* groups (*p < 0.05).

### Administration of *E. coli* expressing IL-35 ameliorates symptoms of DSS-induced colitis

**Fig. 2:**
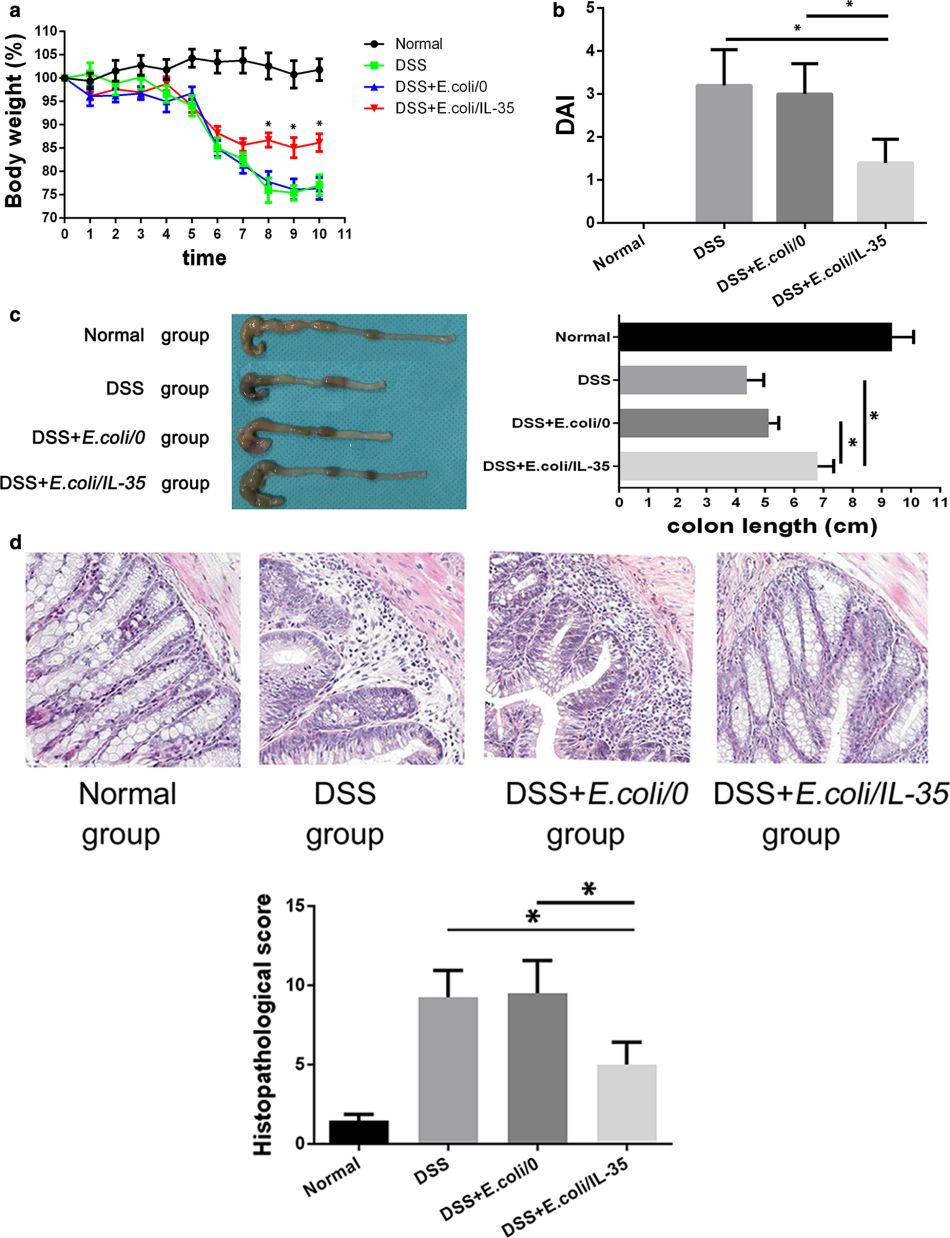
IL-35 ameliorates symptoms of DSS-induced colitis. Body weight (**a**) and disease activity index (DAI) (**b**) change of each group. DSS + *E. coli/IL*-*35* group showed less body weight loss and much lower DAI score. Data were presented as the mean ± SD (n = 10 per group). Compared with the DSS group, *p < 0.05; compared with the DSS + *E. coli/0* group, *p < 0.05. **c** The length of mouse colon from normal group, DSS group, DSS + *E. coli/0* group and DSS + *E. coli*/IL-35 groups. *p < 0.05, *E. coli/IL*-*35* group vs. DSS and DSS + *E. coli/0* group. **d** Representative photomicrographs (×200, haematoxylin and eosin staining) of histological sections of colon from each group. n = 10 mice per group. *p < 0.05, *E. coli/IL*-*35* group vs. DSS and DSS + *E. coli/0* group

DAI score has long been used for evaluating the colon injury. Following induction with DSS, all mice exhibited colitis with persistent liquid or bloody stool, weight loss, and lethargy in all groups before *E. coli* administration. After bacterial treatment, mice in DSS + *E. coli/IL*-*35* group showed significantly less body weight loss (*p < 0.05) (Fig. 2a), firmer stool, as well as food and water consumption with much lower DAI score (*p < 0.05) (Fig. 2b) compared to those in DSS and *DSS *+ *E. coli/0* group. Besides, mice exposed to DSS demonstrated severe intestine shortening and bowel dilation due to intestinal inflammation compared to normal mice. In the DSS + *E. coli/IL*-*35* Group, obvious bowel dilation was not observed, and the length of the colon was significantly longer than that of DSS and DSS + *E. coli/0* group (*p < 0.05) (Fig. 2c). Microscopically, DSS intake caused epithelial injury, while in DSS + *E. coli/IL*-*35* Group, mouse colon presented an improved structure of epithelium and crypts, with much less inflammatory cell infiltration but more regenerative goblet cells. Also, the histopathological scores in DSS + *E. coli/IL*-*35* group were significantly lower than that of DSS and DSS + *E. coli/0* groups (*p < 0.05) (Fig. 2d), suggesting the greatly alleviated injury in the colon following *E. coli/IL*-*35* treatment.

### *E. coli/IL*-*35* suppressed intra-colon infiltration of neutrophils and T cells in the colitis mice

**Fig. 3:**
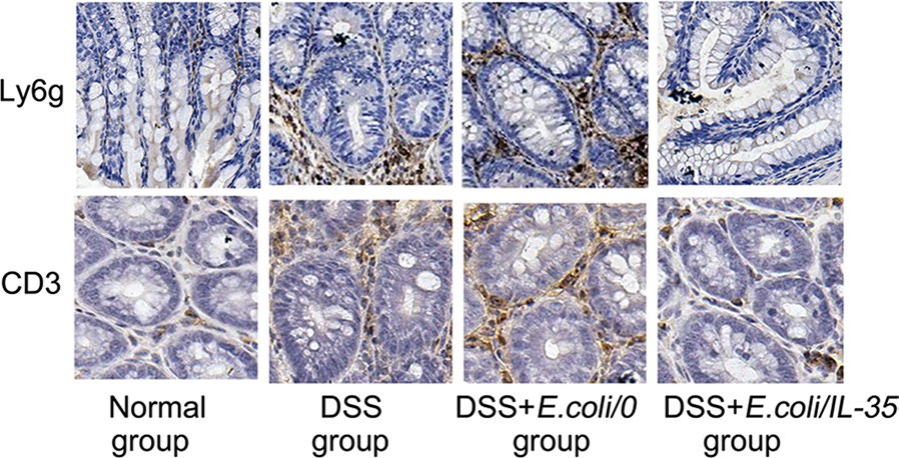
*E. coli/IL*-*35* administration suppressed inflammatory cell infiltration in colon. The neutrophil and T cell accumulations in the colon were examined using antiLy6G and anti-CD3 staining respectively. Representative sections of colon were obtained from normal group, DSS group, DSS + *E. coli/0* group and DSS + *E. coli/IL*-*35* group. Magnification ×100

To investigate the effects of *E. coli/IL*-*35* on attenuation of inflammatory cell infiltration in the colitic mice, we detected and compared intra-colon neutrophil cell and CD3^+^ cell infiltration in all groups. As shown in Fig. 3, neutrophil cell and CD3^+^ cell infiltrations were markedly increased in DSS and DSS + *E. coli*/*0* groups compared with those of normal group and DSS + *E. coli/IL-35* group (*p < 0.05), which demonstrated that administration of *E. coli/IL-35* effectively suppressed neutrophil cell and CD3^+^ cell infiltration.

### Effects of *E. coli/IL*-*35* on spleen and MLN Tregs/Th17 balance

**Fig. 4:**
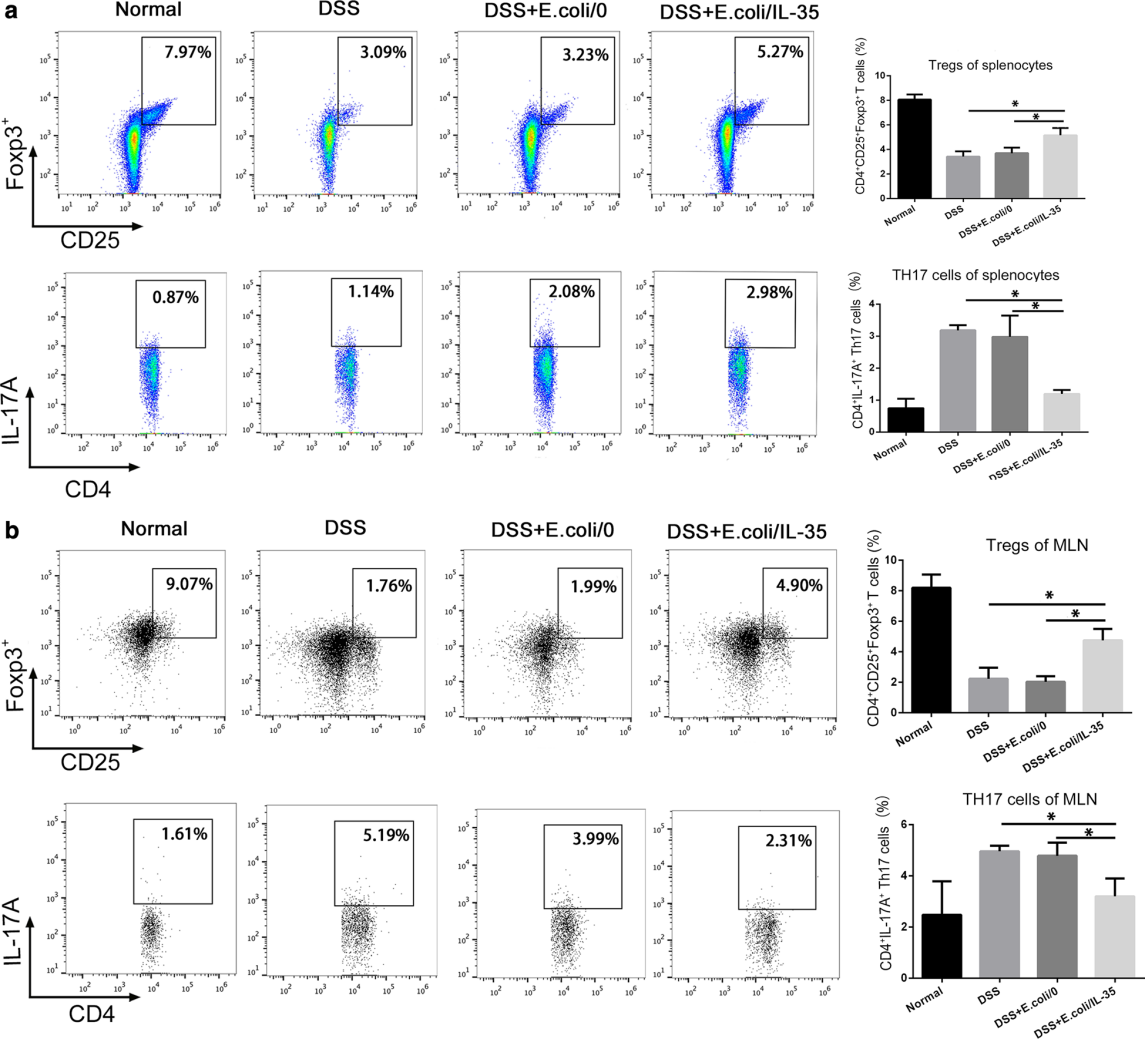
*E. coli/IL*-*35* treatment affected the proportions of Treg and Th17 cells in spleens and MLN. The representative images of flow cytometry for splenocytes and MLN were shown while positive cells were counted from the quadrant Q4. *E. coli/IL*-*35* bacteria increased CD4^+^CD25^+^Foxp3^+^ cells (Treg cells) but reduced IL-17A cells (Th17 cells) in the spleens (**a**) and MLN (**b**) from UC mice, which had lower proportion of Treg cells and higher proportion of Th17 cells as compared to normal controls. Compared with the DSS group, *p < 0.05; compared with the DSS + *E. coli/0* group, *p < 0.05

The effect on Treg/Th17 ratio of IL-35 has been reported, and we also tested the Tregs/Th17 balance in mice spleen and MLN using flow cytometry. As shown in Fig. 4, the levels of CD4^+^CD25^+^Foxp3^+^ Tregs in spleen (Fig. 4a) and MLN (Fig. 4b) were lower in DSS-ingested mice than normal, while the levels of CD4^+^IL-17A^+^ Th17 cells were much higher. Treatment with *E. coli/IL*-*35* significantly enhanced the percentage of Tregs but reduced that of Th17, indicating that *E. coli/IL*-*35* improved the Tregs/Th17 ratio in spleen and MLN.

### Transcription Level of IL-6, IL-10, IL-35 and IL-12 in colon tissues

**Fig. 5:**
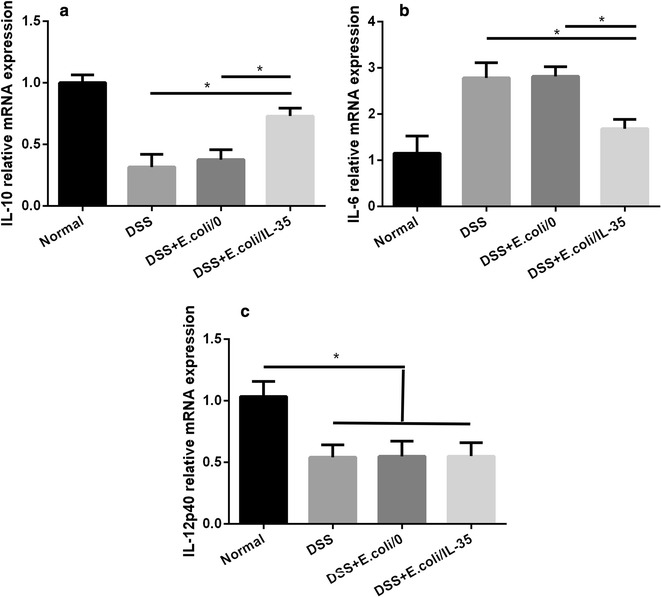
Effects of *E. coli/IL*-*35* on the regulation of IL-10 and IL-6 expressions in colon. The mRNA levels of IL-10 and IL-6 were analyzed by real-time PCR. **a** *E. coli/IL*-*35* treatment increased IL-10 mRNA expression in colon tissues in DSS-induced colitis mice. **b** *E. coli/IL*-*35* intake reduced IL-6 mRNA expression in colon tissues in DSS-induced colitis mice. n = 10 mice per group. Compared with the DSS group, *p < 0.05; compared with the DSS + *E. coli/0* group, *p < 0.05. **c** DSS intake remarkably reduce IL-12p40 expression and *E. coli/IL*-*35* treatment did not change the tendency (*p < 0.01)

To determine whether bacterial treatment would affect the transcriptional levels of cytokines, we measured the intra-colon expression of IL-6, IL-10 and IL-12p40 by real-time PCR. As shown in Fig. 5, after administration of bacterial, the mRNA levels of IL-10 were significantly increased in the DSS + *E. coli/IL*-*35* group compared to that of in the DSS and DSS + *E. coli/0* groups (*p < 0.01) (Fig. 5a). While, no difference between DSS and DSS + *E. coli/0* groups could be observed. DSS ingestion significantly increased the IL-6 level in colitis mice. However, the increase in IL-6 got effectively attenuated in the DSS + *E. coli/IL*-*35* group (*p < 0.01) (Fig. 5b). IL-12p40 level was also detected to exclude the therapeutic potential of IL-12, and the result showed that DSS intake remarkably reduce IL-12p40 expression and *E. coli/IL*-*35* treatment did not change the tendency (*p < 0.01) (Fig. 5c), indicating that IL-35 overexpression contributed more to the benefits post *E. coli/IL*-*35* administration rather than IL-12.

### Effect of *E. coli/IL*-*35* on mouse serum cytokine level

**Fig. 6:**
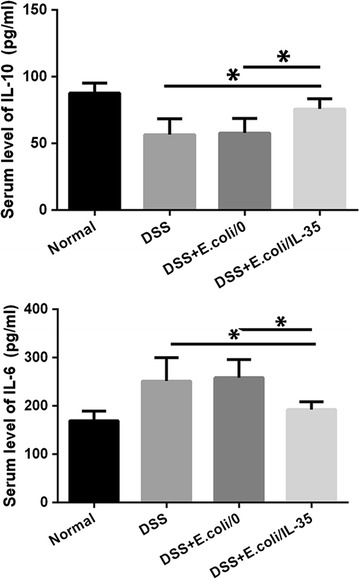
*E. coli/IL*-*35* administration attenuated UC by regulating IL-10 and IL-6 levels in serum. The serum concentration of IL-10 was notably elevated post *E. coli/IL*-*35* treatment. The level of IL-6 was markedly increased in DSS and DSS + *E. coli/0* groups, which got significantly reduced in *E. coli/IL*-*35* group. n = 10 mice per group. Compared with the DSS group, *p < 0.05; compared with the DSS + *E. coli/0* group, *p < 0.05

To determine whether *E. coli/IL*-*35* treatment could affect cytokine profiles, the levels of systemic inflammatory cytokines were analyzed and compared among different groups. The serum concentration of antiinflammatory cytokine IL-10 (Fig. 6) were notably elevated post-*E. coli/IL-35* treatment. Also, the level of a pro-inflammatory cytokine (IL-6) was markedly increased in DSS and DSS + *E. coli/0* groups, which got significantly reduced in *E. coli/IL-35* group (Fig. 6). Taken together, treatment with *E. coli/IL*-*35* not only suppress the level of pro-inflammatory cytokines, but also enhance the level of an anti-inflammatory cytokine in colitis mice.

## Discussion

In the present study, we investigated the therapeutic effects of oral administration of *E. coli/IL*-*35* on DSS-induced experimental colitis in a mouse model. *E. coli/IL*-*35* treatment significantly decreased DAI, attenuated body weight loss, with less intestinal shortening and histopathological changes. Meanwhile, upon treatment with *E. coli/IL*-*35*, the proportion of Tregs increased, and Th17 cells decreased both in spleen and MLN compared with that of DSS group or DSS + *E. coli/0* group, which was also reflected by the correction of cytokine levels in colon tissues. Taken together, these data provided strong evidence that the *E. coli/IL*-*35* treatment significantly affected not only intestinal but also systemic immune responses in UC mice.

UC characterized by chronic and nonspecific inflammation in the large intestine, has been a public problem in western societies. Existing treatments include anti-inflammation drugs and immunosuppressive drugs, which exert a total immunosuppression risk. Recently, analysis of immunoinflammatory pathways in the gut of UC patients has shown that tissue damage is driven by a complex and dynamic crosstalk between immune and nonimmune cells, where cytokines are key mediators of this interplay [18]. The agents targeting tumor necrosis factor (TNF) have been widely acknowledged as the preferred drug in moderating severe UC and CD for more than 15 years [19]. However, systemic distribution to all tissues confers risk of several adverse events, such as infection, lupus-like autoimmunity reactions, and hypersensitivity reactions. Thus, a novel delivery method targeting colon with fewer side effects and higher local drug concentration is needed [7]. Recent studies suggest that engineered intestinal flora can be used as an ideal candidate for local drug delivery.

The bacterial community consists of a complex of microorganism species. *Escherichia coli* is the predominant facultatively anaerobic Gram-negative bacterial species of the normal intestinal flora, in which it plays important roles in promoting the stability of the luminal microbial flora and maintaining normal intestinal homeostasis [20]. Here, a noninvasive *E. coli* BL21 (DE3) expressing a potent anti-inflammatory peptide IL-35 was utilized to provide therapeutic level produced directly in the colon, which was the first use of IL-35 engineered bacteria in colitis treatment. We found a noticeable increase of the cytokine IL-35 among colon specimens in DSS + *E. coli/IL*-*35* group post oral administration (Fig. 1f). Meanwhile, mice treated with *E. coli/IL*-*35* showed significantly reduced weight loss, colon shortening as well as much lower DAI and histological changes (*p < 0.05) (Fig. 2b, d), indicating the effective protection of colon from DSS damage.

Neutrophils and CD3^+^ cell are accumulated in inflamed mucosa of IBD and play an essential role in the pathogenesis [21]. Neutrophils have been found to produce several so-called resolution factors such as lipids mediators [22]. In IBD the tissue damage induced by neutrophils seems higher than normal because of impaired apoptosis and phagocytosis of neutrophils followed by a subsequently extended lifespan [23]. Our study demonstrated that *E. coli/IL*-*35* could remarkably reduce the numbers of Ly6G-positive cells and CD3^+^ cell in the colon compared to that of the untreated group (Fig. 3). Therefore, the beneficial effect on colon exposed to DSS of IL-35 treatment could partly attribute to its suppression of inflammatory cell infiltration.

Much evidence suggests that UC is closely related to the imbalance of Treg/Th17 [24–27]. Tregs mainly function in the suppression of immune responses and maintenance of peripheral tolerance and IBD patients exert lower Tregs level compared to healthy individuals. Th17 cells massively infiltrate the inflamed intestine of IBD patients, where they produce IL-17A and other cytokines, triggering and amplifying the inflammatory process [28]. Thus, Tregs upregulation and/or Th17 suppression may provide a positive intervention on UC or IBD. IL-35 plays an essential role in promoting the optimally suppressive function of Treg cells in vitro and controlling homeostatic proliferation in vivo [9]. Some reports implied that IL-35 could suppress differentiation and function of Th17, rather than TGF-β or IL-10 [10–12]. Systemic delivery of an IL-35 adenoviral vector inhibited disease activity in both DSS and TNBS colitis models [12]. Therefore, bolstering IL-35 induced anti-inflammatory action for therapeutic benefit may be a promising strategy in IBD therapy. We detected the Tregs and Th17 cells of mice splenic and MLN and the results demonstrated that *E. coli*/IL-35 treatment significantly increased the level of CD4^+^CD25^+^Foxp3^+^ Tregs in spleen and MLN compared to that of *E. coli*/0 and PBS group, while the CD4^+^IL-17A^+^ Th17 level got suppressed efficiently (*p < 0.05) (Fig. 4). Interleukins are critical constituents of the cytokine profile participating in intestinal inflammation of IBD patients. The serum and colon levels of pro-inflammatory cytokine IL-6 and anti-inflammatory cytokine IL-10 were measured by Elisa and real-time PCR respectively. The data showed that *E. coli/IL*-*35* administration dramatically reduced the serum IL-6 concentration and increased the IL-10 level, compared to those of untreated mice (*p < 0.05) (Fig. 6). Transcription level analysis of IL-10 and IL-6 in colon tissue got a similar result (Fig. 5a, b). IL-6 has been considered as an essential cytokine for Th17 differentiation, and the lower Th17 cells in *E. coli/IL*-*35* treated mice might be partly related to IL-6 reduction. IL-10 is a well-known tolerogenic cytokine, whose capacity to relieve the intestinal inflammation in UC has been verified [29]. Also, there are reports stated that IL-35 could promote IL-10 secretion and we indeed observed the same result post *E. coli/IL*-*35* treatment. These findings implied that local administration of IL-35 by recombinant *E. coli* effectively intervened in the pathological inflammation caused by DSS exposure and the positive effect was partly due to the immune homeostasis improvement in the colon and whole body.

## Conclusion

The present study tested the efficacy of recombinant bacteria *E. coli/IL*-*35* in the colitis mice post oral administration. The data indicated that *E. coli/IL*-*35* treatment significantly affected not only intestinal but also systemic immune responses in UC mice. In conclusion, *E. coli*, as a novel delivery system to deliver IL-35, provides an alternative strategy for DSS-induced ulcerative colitis therapy. However, further studies are still needed to elucidate the complex pathways underlying *E. coli/IL*-*35*-mediated colon protective effects.

## Abbreviations

UC: ulcerative colitis
IBD: inflammatory bowel disease
IL: interleukin
DSS: dextran sulfate sodium
*E. coli*: *Escherichia coli*
EBI3: Epstein–Barr virus-induced gene
IPTG: isopropyl-β-d-thiogalactopyranoside
DAI: daily disease activity index
Ly6G: lymphocyte antigen 6 complex, locusG
MLN: mesenteric lymph nodes
Treg: regulatory T cell
TNF: tumor necrosis factor
TGF-β1: transforming growth factor-β1
